# Hemophagocytic Syndrome and COVID-19: A Comprehensive Review

**DOI:** 10.7759/cureus.36140

**Published:** 2023-03-14

**Authors:** Mahdi M Fadlallah, Sarah M Salman, Mariam M Fadlallah, Hassan Rahal

**Affiliations:** 1 Department of Laboratory Medicine, Faculty of Medical Sciences, Lebanese University, Beirut, LBN; 2 Department of Laboratory Medicine, Al-Zahraa Hospital University Medical Center, Beirut, LBN; 3 Department of Pediatrics, Bahman Hospital, Beirut, LBN; 4 Department of Infectious Diseases, Bahman Hospital, Beirut, LBN

**Keywords:** cytokine storm, immune system, covid-19, haemophagocytic lymphohistiocytosis, haemophagocytic syndrome

## Abstract

Hemophagocytic lymphohistiocytosis (HLH), a hyperinflammatory hyperferritinemic syndrome, is triggered by various etiologies and diseases and can lead to multiorgan dysfunction and death. There are two types of HLH: primary and secondary. Primary HLH (pHLH) is caused by a genetic mutation resulting in dysfunction in cytotoxic T lymphocytes (CTLs), natural killer (NK) cells, hyperactivated immune cells, and hypercytokinemia. In secondary HLH (sHLH), an underlying etiology is the cause of the disease. Infections, malignancy, and autoimmune diseases are well-known triggers for sHLH. Infectious triggers for sHLH are most frequently viruses, where different mechanisms, including dysregulated CTLs and NK cell activity and persistent immune system stimulation, have been reported. Similarly, in severe coronavirus disease 2019 (COVID-19) patients, a hyperinflammatory mechanism leading to hypercytokinemia and hyperferritinemia has been demonstrated. A similar dysfunction in CTLs and NK cells, persistent immune system stimulation with increased cytokines production, and severe end-organ damage have been reported. Therefore, a significant overlap is present between the clinical and laboratory features seen in COVID-19 and sHLH. However, SARS-CoV-2, similar to other viruses, can trigger sHLH. Hence, a diagnostic approach is needed in severe COVID-19 patients presenting with multiorgan failure, in whom sHLH should be considered.

## Introduction and background

Cytokine storm (CS) and cytokine release syndrome (CRS) are potentially fatal, similar syndromes of systemic inflammation resulting from immune dysregulation. Among others, hemophagocytic lymphohistiocytosis (HLH), sepsis, autoinflammatory disorders, and recently coronavirus disease 2019 (COVID-19) have been described as causes of CS and CRS. The high levels of circulating cytokines combined with the hyperactivated immune system seen in CS/CRS can be triggered by various causes, including but not limited to monogenic disorders and pathogens. This will lead to potentially fatal systemic and dysregulated inflammation, resulting in multiorgan dysfunction and failure [1,2].

HLH is a rare, potentially fatal condition characterized by a hyperinflammatory CS caused by a dysregulated, excessively activated, and ineffective immune system [2,3]. Hypercytokinemia causes persistent fever, cytopenias, and multiorgan dysfunction [4,5]. The spleen and the liver are the most common organs involved. In addition, respiratory distress syndrome and renal failure are common [5]. HLH should be investigated in patients with unexplained fever, cytopenias, and multiorgan failure, including acute respiratory distress syndrome (ARDS), renal failure, and neurological impairment [6].

In COVID-19 patients, clinical symptoms severity ranges from mild or totally asymptomatic patients to severe illness, with ARDS, multiorgan failure, and death [7,8]. The disease severity and mortality in COVID-19 were mainly attributed to the hyperinflammatory state with the subsequent CS resulting in clinical features reminiscent of those seen in HLH [7,9].

Thus, there are significant clinical and laboratory similarities between COVID-19-induced CS and HLH [10]. In addition, a link between severe COVID-19 illness and HLH has been proposed [11].

**Figure 1:**
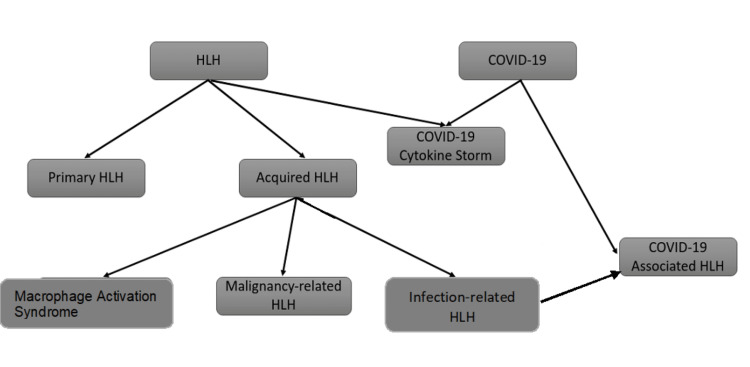
HLH variants and COVID-19 Note. This figure summarizes the different types of HLH. In addition, it shows the COVID-19 cytokine storm as a variant of HLH and the COVID-19-associated HLH as a part of the acquired HLH (infection-related HLH). HLH: hemophagocytic lymphohistiocytosis.

In this review, we aim to elucidate the overlap between the hyper-inflammatory state and CS in severe COVID-19 and the HLH syndrome in terms of pathophysiology and clinical manifestations (Figure 1).

## Review

HLH

HLH is a hyperinflammatory hyperferritinemic syndrome, caused by severe hypercytokinemia and an inherited or acquired inability of the immune system to respond to a trigger, which, in most cases, has an infectious origin [3,4]. When there is a positive family history or a defined genetic cause, it is classified as genetic or primary HLH (pHLH) [12].

Primary HLH

Genetic HLH can be categorized into two groups: familial HLH (F-HLH) and those related to primary immune deficiency syndrome [13]. Genetic HLH is caused by a loss of function of cytotoxic T cells and natural killer (NK) cells due to inherited genetic mutations in a homozygous or compound heterozygous pattern [9]. Various mutations in at least nine genes are involved in pHLH [12]. Among these mutations, the PRF1 gene mutation is the most common, followed by UNCD13D, STX11, and STXBP2 [9,14]. Immunodeficiency syndromes with mutations affecting the RAB27A, LYST, SH2D1A, and BIRC4 genes frequently present with HLH either as the initial presentation of the disease or later on during the course of the disease [14]. All affected genes have a role in cytotoxic granules exocytosis or function [3,9]. Cytotoxic granules in the cytotoxic T cells and NK cells contain perforins and granzymes [2,9]. These granules are released normally in response to a trigger, usually a virus or tumor cells. Their release between cytotoxic cells and target cells occurs through an immunological synapse [2,9]. Perforins cause osmotic lysis of the target cell through pore formation and allow granzymes entry into the target cell, resulting in apoptotic cell death [9,15]. The apoptosis of the target cells and the clearance of the antigenic stimulus cause immune response termination [15]. Any defect in perforins or cytotoxic granules exocytosis will result in an inability to clear an antigenic stimulus with persistent stimulation of T cells and subsequent macrophage activation [15]. Consequently, abnormally high levels of proinflammatory cytokines such as interleukins (ILs) IL-2, IL-6, IL-12, IL-16, and IL-18, interferon-gamma (IFN-δ), and tumor necrosis factor-alpha (TNF-α) are observed [9,16]. The majority of patients with pHLH are diagnosed in the first year of life [9].

Acquired HLH

In secondary HLH (sHLH), the majority of cases occur in adults [17]. Various underlying conditions are associated with the disease. Autoimmune diseases, malignancy, and infection are the predominant triggers [14,17]. When occurring in autoimmune disease, it is termed "macrophage activation syndrome" (MAS) and is mostly found in adult-onset Still’s disease, systematic juvenile idiopathic arthritis, and systemic lupus erythematosus (SLE) [3]. In malignancies associated with sHLH, T-cell-derived lymphoma and leukemia are the most reported triggers [3,14].

Infection-related HLH: HLH triggered by infections is mostly caused by viruses [17]. Bacterial infections are rarely reported as triggers for HLH, and the majority of reported cases are caused by mycobacterial tuberculosis [18]. Human herpes virus (HHV), Epstein-Barr virus (EBV), and cytomegalovirus (CMV) are among the most common viruses associated with HLH. However, cases of influenza (H1N1 and H5N1), parvovirus B19, and hepatitis A and C-associated HLH have been reported [18,19]. Various mechanisms have been described as possible drivers for HLH in viral infections [20]. In chronic viral infections, a sustained stimulation of innate immunity may result in excessive and chronic activation of the toll-like receptors 9 (TLR9), which was shown to be linked to HLH/MAS-like syndrome in mouse models [20,21]. This process, however, has been observed in parasitic (visceral leishmaniasis) and bacterial infections (*Mycobacterium tuberculosis, Salmonella typhimurium*), in which histiocytes containing persistent pathogens continuously activate toll-like receptors (TLRs) [14]. In addition, the viral infection of the immune cells impairs their function [20]. Cytotoxic T lymphocytes (CTLs) infection was predominant in EBV-associated HLH [22] and resulted in possible immortalization and persistent cytokine production [20]. Moreover, inhibition or delay of apoptosis through various anti-apoptotic proteins in hepatitis C virus (HCV), EBV, HHV-8, and adenovirus was reported [20]. Thus, unsuccessful induction of apoptosis and prolonged target cell survival may result in amplified quantities of inflammatory and proinflammatory cytokine secretion from CTLs/NK lymphocytes and macrophages, resulting in a CS [23]. Furthermore, CTLs and NK cells' cytotoxic functions were impaired in various viral infections [20]. Impaired cytolytic response of CTLs to antigen-presenting B cells due to defective signaling lymphocyte activation molecule-associated protein (SAP) expression in EBV [24,25], as well as reduced perforin expression in CTLs in H5N1 influenza virus [26], were reported. NK cells are essential for HLH resistance and viral control [20]. Thus, a decline in NK cells number, reported in several viral infections, favors the development of HLH [20]. As well, several DNA viruses encode cytokines, binding proteins, chemokines, and cellular growth factors and may potentially disturb cytokine balance, disrupt immune homeostasis, and contribute to a CS [20]. Finally, the detection of monoallelic sequence variation within the genes implicated in familial HLH in a significant number of patients with adult-onset HLH may play a role in disease development, namely, when challenged by viral infections [27,28].

Malignancy-related HLH (M-HLH): In the context of malignancy, M-HLH accounts for approximately half of adult HLH cases [29]. M-HLH can occur either during or before the treatment of a diagnosed malignancy. However, M-HLH can also present as the first manifestation of malignancy if not previously diagnosed [30]. Hematological malignancies, including T/NK cell disorders, lymphoma, acute leukemia, lymphoproliferative, and myelodysplastic diseases, are frequently reported as triggers for M-HLH and are mostly reported in adults [18,31]. A worse prognosis is reported in M-HLH compared to other congenital or acquired HLH [5]. The immunodeficient state in malignant patients, linked to the malignancy itself or the chemotherapy, increases susceptibility to infections and T/NK cell dysfunction and lowers the threshold for triggering HLH in these patients [31,32]. Moreover, treatment with immunotherapies can result in a CS that is clinically and immunologically similar to HLH, through the overproduction of proinflammatory cytokines by the immunotherapy-activated T-cells [31].

MAS: MAS is the terminology for sHLH occurring in autoimmune diseases. The prevalence of MAS in autoimmune diseases has been mostly reported in systematic juvenile idiopathic arthritis (sJIA) and SLE [33,34]. Although, MAS has been reported in other autoimmune diseases, including but not limited to rheumatoid arthritis [35], sarcoidosis, Crohn’s disease, and polymyalgia rheumatica [36]. Similar to other types of HLH, impaired T/NK cell function is presumably the underlying mechanism for MAS development, although dysregulation in NK cells has been implicated in the pathogenesis of autoimmune diseases [37]. In addition, decreased expression of perforin and SAP genes has been reported in MAS patients [13]. However, a high level of suspicion is required to detect MAS in an autoimmune disease since several features of MAS can be shared with the underlying immune disorder, such as hyperferritinemia in sJIA and cytopenias in SLE [33].

HLH clinical features, diagnosis, and treatment

Clinical Features

HLH is considered a medical emergency, and suspicion should be raised in cases presenting with signs and symptoms of systemic inflammatory response syndrome (SIRS) in the absence of any underlying causes [13]. Fever, organomegaly (hepatomegaly, splenomegaly, and/or lymphadenopathy), and cytopenias are among the most typical findings in HLH patients [13,38]. Moreover, neurological involvement symptoms (seizures, ataxia, hemiplegia, etc.), coagulopathy symptoms (purpura, petechiae, hemorrhage, and disseminated intravascular coagulation (DIC)), and a nonspecific rash have been reported [9,13]. In neonates, the clinical presentation may differ, with absent fever in most cases. Accordingly, the presence of hepatomegaly, coagulopathy, and cytopenias in these patients should raise suspicion for HLH, even in the absence of fever [13].

Diagnosis 

The 2004 revised diagnostic guidelines for HLH (HLH-2004) are commonly used in diagnosing and scoring HLH [9]. HLH-2004 requires either a molecular diagnosis of HLH or the presence of five out of eight clinical and laboratory criteria. These include fever, splenomegaly, cytopenias affecting two or more cell lines in the peripheral blood, hypertriglyceridemia and/or hypofibrinogenemia, elevated ferritin, low or absent NK cell activity, elevated soluble IL-2 receptor (sIL-2R), and evidence of hemophagocytosis in the bone marrow, lymph nodes, or spleen [38].

Another diagnostic scoring system that has been widely used is the HLH-probability calculator (H-score). It includes nine variables (three clinical, five biological, and one cytological) included in the development of HLH: the presence of underlying immunosuppression, fever, organomegaly, cytopenia, elevated ferritin level, triglycerides level, fibrinogen level, aspartate aminotransferase/serum glutamic oxaloacetic transaminase level, and hemophagocytosis features in bone marrow aspirate [9,39]. Each criterion has a weight in the scoring system [39]. A total score ranging from zero to 337 can be calculated. The optimal cutoff for diagnosing HLH was shown to be 169 (Table 1) [38,39]. Debaugnies et al. compared the two diagnostic systems and discovered that the H-score is less restrictive and more predictive for diagnosing HLH than the HLH-2004 diagnostic guidelines [40].

**Table 1:** Clinical and laboratory findings included in the adapted H-score and HLH-2004 guidelines * Diagnosis requires either the presence of molecular diagnosis or the presence of five out of eight criteria. ** A scoring cutoff of 169 is used to diagnose HLH. HLH: hemophagocytic lymphohistiocytosis; NK: natural killer; IL-2: interleukin-2.

Clinical/laboratory finding	HLH-2004*	H-score**
Fever	>38.5°C	<38.4: 0 points
		38.4-39.4: 33 points
		>39.4: 49 points
Organomegaly	Splenomegaly	Absent: 0 points
		Hepatomegaly OR splenomegaly: 23 points
		Hepatomegaly and splenomegaly: 38 points
Cytopenia	≥2 of 3 lineages in peripheral blood	One lineage: 0 points
Hemoglobin < 9 g/dL		Two lineages: 24 points
Platelet count < 100*10⁹/L		Three lineages: 34 points
Neutrophils < 1.0*10⁹/L		
Fibrinogen	<1.5 g/L	>2.5 g/L: 0 points
		≤2.5 g/L: 30 points
Triglycerides	>3.0 mmol/L (>265mg/dL)	<1.5 mmol/L: 0 points
		1.5-4 mmol/L: 44 points
		>4 mmol/L: 64 points
Ferritin	>500 µg/L	<2,000 µg/L:0 points
		2,000-6,000 µg/L: 35 points
		>6,000 µg/L: 50 points
Hemophagocytosis in bone marrow	Hemophagocytosis in bone marrow liver, spleen, lymph nodes, or other tissues	Absent: 0 points
		Present: 35 points
NK cells activity	Low or absent	N/A
Soluble CD25 (soluble IL-2 receptor)	≥2,400 U/mL	N/A
Known underlying immunosuppression	N/A	Absent: 0 points
		Present: 18 points
Serum aspartate aminotransferase (AST)	N/A	<30 U/L: 0 points
		>30 U/L: 19 points

HLH Treatment

The main goals of HLH treatment are to eliminate the triggers, the activated immune cells, and antigen-presenting cells and to suppress hyperinflammation. The HLH-2004 protocol was designed for HLH treatment regardless of the evidence for F-HLH or viral infections [38]. Primarily, the protocol consists of eight weeks of initial therapy with etoposide, cyclosporine A, dexamethasone, treatment of possible triggers, and supportive care using broad-spectrum antibiotics, antifungals, antiviral therapy, and intravenous immunoglobulin (IVIG) [14,38]. Further continuation therapy after the initial phase is required in patients with familial history of HLH, patients with persistent disease, and those with disease reactivation after the initial therapy. It consists of pulses of etoposide and dexamethasone [38,41]. An additional intrathecal therapy using intrathecal methotrexate and hydrocortisone is recommended in patients with signs of persistent CNS involvement and CNS reactivation [38,41]. Additional treatment protocols have been reported as salvage therapies in refractory cases that do not respond to treatment and relapse before hematopoietic stem cell transplantation (HSCT). Reports describing the use of alemtuzumab, infliximab, daclizumab, anakinra, and other agents exist in the literature [41]. Finally, HSCT is indicated in cases of refractory HLH, CNS involvement, and F-HLH for the elimination of the immune defect [14].

COVID-19 cytokine storm

Pathogenesis

**Figure 2:**
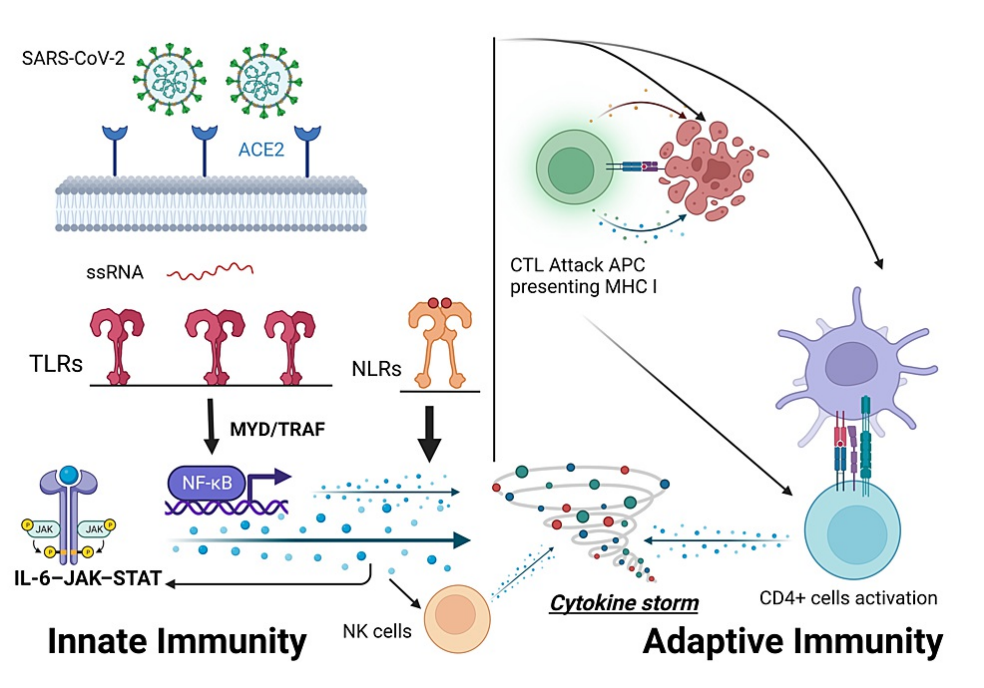
Pathogenesis of the COVID-19 cytokine storm After binding to ACE2 receptors and entering the host cell, ssRNA is recognized by the innate immune system recognition receptors, including the toll-like receptors (TLRs). This results in the activation of NF-ĸB and the release of pro-inflammatory cytokines through different pathways (e.g., MYD and TRAF). The adaptive immune cells recognize pathogens and infected cells through MHC I and II present on cytotoxic T lymphocytes (CTLs) and CD4+ T cells, respectively. Activation of the adaptive immune system results in an amplified inflammatory reaction, which results in additional pro-inflammatory cytokines release. In addition, NK cells activated by the innate infected cells cytokines participate in the pathogenesis of the cytokine storm by the secretion of other regulatory cytokines that activate other immune pathways. ACE2: angiotensin-converting enzyme 2; ssRNA: single-stranded ribonucleic acid; MHC: molecular histocompatibility complex; NK: natural killer; NLRs: NOD-like receptors; APC: antigen-presenting cells. Note. This figure was created with BioRender.com.

The CS seen in HLH has been described in patients with severe COVID-19 disease [42]. A high level of proinflammatory cytokines was demonstrated in severely ill patients with COVID-19 compared to those with a milder disease course, indicating a worse prognosis and increased mortality [43]. Severe COVID-19 illness is associated with a cytokine profile similar to that seen in sHLH, including increased interferon-induced protein 10 (IP-10), monocyte chemoattractant protein 1 (MCP-1), macrophage inflammatory protein (MIP) 1, IL-2, IL-6, IL-7, granulocyte colony-stimulating factor (G-CSF), and TNF-α [44,45]. Critical COVID-19 illness has been linked to an overactive innate and adaptive immune system producing high levels of proinflammatory cytokines [46]. SARS-CoV-2 binds with high affinity to the angiotensin-converting enzyme 2 (ACE2) receptor, which is considered the gate for its single-stranded ribonucleic acid (ssRNA) entry into the host cells. The ssRNA is recognized by innate immune recognition receptors such as TLRs. It induces the activation of the nuclear factor kappa light chain enhancer of activated B cells (NF-ĸB) pathway and results in the release of several pro-inflammatory cytokines through different pathways (MYD88, TRAF, and JAK/STAT, etc.) and the amplification of the anti-inflammatory process. Furthermore, TLRs activation results in the release of type I interferon, which has a direct antiviral activity and activates the adaptive immune system [46-48]. Additionally, the NOD-like receptors (NLRs) sense the damage-associated molecular pattern (DAMP), released from dying and damaged cells, resulting in high levels of IL-1β, one of the main activators of IL-6 [46,49]. The adaptive immune cells recognize pathogens and infected cells through molecular histocompatibility complex (MHC) classes I and II, identified by CD8+ cytotoxic T cells (CTLs) and CD4+ T cells, respectively. MHC class II molecules are present on the surface of the antigen-presenting cells (APCs) membrane. APCs are monocytes, macrophages, and dendritic cells and they activate CD4+ T cells and B cells. MHC class I molecules are present on the surface of all nucleated cells [47]. CTLs kill the APC and result in an amplified inflammatory reaction through further CD4+ T cells amplification, which results in an additional release of pro-inflammatory cytokines (IL-6, IFN-δ, and granulocyte-macrophage colony-stimulating factor (GM-CSF)) and further macrophage activation [46,50]. Moreover, NK cells activated by the innate infected cells cytokines eradicate viruses through either their cytolytic activity on target cells or the secretion of other regulatory cytokines that activate other immune pathways, namely, TNF-α and IFN-δ (Figure 2) [47,50,51]. The previously mentioned inflammatory state in COVID-19 is biphasic. The first phase is characterized by cytokines secretion by the infected airway epithelial cells, airway macrophages, and other innate immunity cells. This results in the further attraction of innate and adaptive immune cells, including macrophages, dendritic cells, CD4+ cells, and NK cells, and leads to the second wave of cytokines secretion and further infiltration of lungs with immune cells. This second wave typically occurs within seven days of infection and includes IFN-γ, TNF-α, IL-2, IL-5, IL-6, and chemokines [50]. The CS in COVID-19 can also result from an impaired immune system [4]. An impaired NK cell cytolytic activity as well as reduced counts were linked to the hyperinflammation seen in severe COVID-19 patients [52]. The high levels of IL-6, being associated with severe COVID-19, have been shown to reduce the expression of perforin and granzyme B in NK cells, subsequently reducing their cytolytic activity [48,53]. Mazzoni et al. found an inverse correlation between NK cells’ cytotoxic potential and high levels of IL-6 [54]. Additionally, an upregulation of the inhibitory NK cell receptor NKG2A in COVID-19 patients was associated with exhausted, hyporesponsive NK and CTLs with reduced cytotoxic potential as well as reduced IFN-γ levels [55]. Also, a depletion of CD56^dim^ and CD56^bright^ NK cells, involved in cell-mediated cytotoxicity and production of cytokines (IFN-γ and TNF-α), has been reported in severe COVID-19 patients [56]. Consequently, an inadequate antiviral response in the early stage of infection due to a reduced interferon response and reduced cytolytic potential may result in delayed viral clearance, leading to continuous immune stimulation and amplified concentrations of pro-inflammatory cytokines [1,4,50]. Furthermore, another mechanism was hypothesized in pediatric patients with post-infection multi-system inflammatory syndrome. An autoimmune reaction caused by molecular mimicry between self-antigen and SARS-CoV-2 has been suggested [1].

Treatment

The treatment of the CS seen in COVID-19 is aimed to control the inflammatory reaction and prevent further organ damage through targeted immunosuppression [57]. The efficacy of antiviral therapy with remdesivir has been reported to shorten the course of disease in hospitalized COVID-19 patients [58]. Although, only the anti-inflammatory approaches, using immunomodulators, have shown to improve survival in these patients [59]. Corticosteroids including dexamethasone have an anti-inflammatory activity against various cytokines (IFN-δ, TNF-α, IL-1, IL-2), and have been shown to reduce mortality in patients requiring respiratory support [48,57]. Appropriate timing and dosing for corticosteroids are important and should only be administered to patients presenting with clinical, radiological, and laboratory markers suggestive of a CS [60]. Dexamethasone administration to hospitalized patients with COVID-19 was studied in the RECOVERY trial. A significant decrease in the 28 day-mortality was observed in patients receiving respiratory support [61].

Cytokine targeting and suppression could result in decreased proinflammatory signaling and improved clinical status [57]. Inhibition of IL-6, a key mediator of the inflammatory process in CS and a prognostic marker in COVID-19, with IL-6 receptor inhibitors including tocilizumab and sarilumab has shown its efficacy in the severe COVID-19 stages [1,48]. Earlier studies on tocilizumab had varying results and effectiveness was demonstrated in multiple studies [62-64], while others failed to show any clinical improvement in patients using tocilizumab compared to standard care [65]. Later randomized controlled trials (RCTs) showed improvement in clinical outcomes in severe COVID-19 patients using tocilizumab [66-68]. A larger RCT conducted by the RECOVERY group enrolled 4,116 patients with hypoxia and systemic inflammation. An increased survival, a reduction of disease progression to invasive ventilation, and increased chances of successful hospital discharge were all reported with the use of tocilizumab [69]. Sarilumab, another IL-6 inhibitor, was studied in the REMAP-CAP study and showed benefits in severe COVID-19 patients [66]. At present, the Infectious Diseases Society of America (IDSA) recommends the use of tocilizumab in critically ill COVID-19 patients with systematic inflammation in combination with standard of care, including steroids. If not available, sarilumab can be used instead of tocilizumab [48]. IL-1, a cytokine generated by macrophages, activating neutrophils that become uncontrolled and result in severe lung injury, ARDS, and multiorgan failure, was studied as a target for inhibition with the monoclonal antibody anakinra [48,70]. Anakinra, a drug used in some autoinflammatory diseases and familial Mediterranean fever (FMF), blocks IL-1 alpha and beta at the IL-1 receptor, thus inhibiting IL-1 pro-inflammatory effects [60]. A prospective cohort study conducted by Huet et al. demonstrated reduced mortality and the need for mechanical ventilation in patients with severe COVID-19 disease [71]. Similarly, improved survival in patients with COVID-19-associated ARDS was shown in another retrospective study [72]. On the other hand, a multicenter, open-label, Bayesian randomized clinical trial (CORIMUNO-ANA-1) did not show any associated clinical improvement in COVID-19 pneumonia patients who received anakinra [73]. Currently, anakinra is not yet approved for COVID-19 treatment [48,74].

Additionally, inhibition of the IL-6-JAK-STAT3 pathway has been suggested to reduce cytokine production [75]. The use of baricitinib in conjunction with remdesivir showed a shorter recovery time compared to remdesivir alone [76]. In addition, a larger study (COV-BARRIER) evaluated the efficacy of baricitinib in critically ill COVID-19 patients. A significant reduction in mortality was observed in patients receiving baricitinib compared to the placebo group [77]. A recent meta-analysis of RCTs evaluating the effectiveness of baricitinib showed evidence of improved mortality in COVID-19 patients receiving baricitinib in conjunction with standard of care [78]. Currently, the only FDA emergency use authorization-approved JAK inhibitor is baricitinib [48].

GM-CSF, a pro-inflammatory cytokine secreted by immune cells, including T cells and macrophages, has been suggested as a potential driver for inflammatory lung injury and subsequent ARDS in severe COVID-19 patients [48]. Agents that interfere with GM-CSF action have been suggested to reduce the inflammatory response and prevent lung injury in severe COVID-19 patients [48,79]. A monoclonal neutralizing antibody against GM-CSF, lenzilumab, has been reported to improve survival in ventilator-free patients in a multicenter RCT [80]. Other GM-CSF inhibitors, including otilimab and mavrilimumab, have been studied and showed no clinical or survival benefit for patients receiving GM-CSF compared to the placebo group [81,82].

The COVID-19 Treatment Guidelines Panel recommends the use of dexamethasone, tocilizumab (or sarilumab), and baricitinib as immunomodulators in hospitalized COVID-19 patients. Regarding anakinra and other immunomodulators such as GM-CSF inhibitors, the panel does not recommend for or against their use in COVID-19 patients due to insufficient evidence [83].

Tocilizumab and anakinra have been shown to be effective in sHLH, drug-induced HLH, and MAS, respectively [84].

COVID-19: sHLH variant?

Since severe COVID-19 and HLH share similar clinical and pathophysiological features, the rapid disease progression observed in COVID-19 patients can be related to sHLH developing in response to viral infection [11]. Thus, the H-score, an HLH diagnostic scoring system, was used in various studies to compare the two diseases as well as a possible tool to assess prognosis in severely ill patients [42,85-89]. A study conducted by Hakim et al. compared the clinical features seen in sHLH with those seen in the COVID-19 CS using the H-score diagnostic system. Most of the severe COVID-19 infections did not reach the H-score cutoff used in diagnosing HLH [42]. Comparably, Wood et al. found a low number of patients with severe COVID-19 illness having an H-score suggestive of HLH [90]. Similarly, Loscocco et al. reported the absence of a correlation between the H-score and COVID-19 severity [88]. A similar low median H-score and low incidence of HLH were found among hospitalized COVID-19 patients [91]. This was mainly attributed to the absence of hepatomegaly, hypofibrinogenemia, cytopenias, and lower elevations of ferritin levels [42,88,90,91]. In addition, splenomegaly was less frequently reported in COVID-19 patients in comparison to HLH patients [4]. Also, the high D-dimer levels seen in HLH as a result of DIC were attributed to pulmonary microthrombosis in severe COVID-19 patients [92]. Moreover, a comparison of serum biomarkers between sHLH and COVID-19 revealed different profiles. Reduced levels of soluble Fas ligand, as well as lower IL-18 and IFN-γ, were seen in the course of COVID-19 infection compared to sHLH or MAS. In contrast, increased serum levels of IL-1 receptor antagonist, IL-8, and intercellular adhesion molecule 1 are seen in severely critical COVID-19 patients [93]. Furthermore, lower levels of NK cell activating cytokines, namely, IL-12, IL-15, and IL-21, were found in hospitalized COVID-19 patients compared to sHLH and suggested a different pattern of NK cells' suppression [52]. Additionally, it has been suggested that ARDS developing in COVID-19 pneumonia patients is a manifestation of localized sHLH in the lungs [92], where the high levels of inflammatory markers such as C-reactive protein, ferritin, lactate dehydrogenase, and IL-6 reflect inflammatory lung damage rather than systematic macrophage activation [88]. However, the localized severe lung injury can lead to increased pulmonary endothelial permeability, shedding of the virus, and other pathogen-associated molecular pattern molecules into the systematic circulation, and activation of a systematic inflammatory response that can lead to amplified cytokine production and multiorgan failure. TNF-α, IL-1β, and IL-6 systematic release from alveolar macrophages and airway epithelial cells can lead to monocytes, macrophages, and T cells hyperactivation and result in sHLH [94]. Accordingly, involvement of the reticuloendothelial organs was evident in various studies and hemophagocytosis was reported in the bone marrow and the spleen [95-97].

COVID-19-associated HLH

HLH is a highly fatal disease, and early management is required [98]. Despite being rare, assessment of the H-score was suggested to be useful for detecting suspected HLH in COVID-19 patients [11,87]. In contrast to the earlier mentioned reports [85,88,91,99], a high incidence of sHLH in severely ill COVID-19 patients was shown in other studies (Table 2) [42,86-88,90,95,96]. Furthermore, various reports of sHLH in recovered COVID-19 patients have been made [10,99-102]. A more severe disease progression and higher mortality were seen in patients with higher H-scores and suspected HLH [86,87,91]. Among the previously mentioned studies, Meng et al. identified three risk factors for differentiating sHLH in COVID-19 patients. Thrombocytopenia (<101x10⁹/L), elevated ferritin (>1922.58 ng/ml), and triglycerides level (>2.28 mmol/L) were identified as independent risk factors for sHLH in COVID-19 patients [87].

**Table 2:** H-score parameter frequencies and sHLH/MAS incidence based on diagnostic H-score > 169 among COVID-19 patients in seven different studies AST: aspartate aminotransferase; sHLH: secondary hemophagocytic lymphohistiocytosis; MAS: macrophage activation syndrome.

H-score parameter	Hakim et al. [42]	Allen et al. [86]	Loscocco et al. [88]	Wood et al. [90]	Núñez-Torrón et al. [95]	Dandu et al. [96]	Ruscitti et al. [89]
Fever	2/14	147/242	7/48	36/40	12/16	13/13	40/47
Organomegaly	1/14	36/242	11/48	0/40	4/16	1/13	13/47
Ferritin > 2,000 ng/mL	4/14	75/242	6/48	24/40	4/16	5/13	6/47
Triglycerides > 133 mg/dL	5/14	130/242	65/48	32/33	Plate	9/13	23/47
Hemophagocytosis	N/A	N/A	N/A	0/1	16/16	13/13	0/13
Cytopenia (2 or more lineage)	0/14	83/242	4/48	14/40	11/16	5/13	8/47
Fibrinogen < 250 mg/dL	0/14	9/242	7/48	2/40	11/16	3/13	3/47
Immunosuppression	1/14	14/242	2/48	3/40	2/16	0/13	0/47
AST > 30 U/L	13/14	232/242	26/48	40/40	16/16	10/13	20/47
Total H-score > 169	1/14 (7.14%)	32/242 (13.2%)	0/48 (0%)	3/40 (7.5%)	10/16 (62.5%)	4/13 (30.76%)	5/47 (10.63%)

Immune dysregulation and an unrecognized persistent inflammatory state appear to play a role in the pathogenesis of COVID-19-associated HLH [100]. As mentioned earlier, a reduced number of NK cells, along with lymphopenia and impaired NK cytolytic function, were evident in COVID-19 patients [103]. Moreover, the characteristic activated T cell (CD38 high) expansion seen in sHLH, which differentiates it from sepsis [104], has been demonstrated in severe COVID-19 and was associated with systemic inflammation and tissue injury [105]. Thus, the suppression of NK cell function along with the persistent activation of T cells may potentiate the development of sHLH in COVID-19 [105,106]. Furthermore, innate immune system overactivation through TLRs and subsequent activation of Janus kinase transducers (JAK/STAT), previously reported in the pathogenesis of HLH and mentioned earlier in COVID-19 pathogenesis, may lead to sHLH in COVID-19 patients [107,108]. On top of the above factors, the presence of monoallelic sequence variation in the genes involved in F-HLH and primary immunodeficiency disease may play a role in the severity of COVID-19 and the development of HLH. The presence of a heterozygous variant of the PRF1 gene (A91V PRF1) implicated in F-HLH was reported in two out of 22 COVID-19 patients. Patients harboring the gene variant experienced a more severe illness, had an H-score above the cut-off point (>169), and both died [109]. Similarly, Luo et al. identified variants in four genes involved in primary immunodeficiency disease and F-HLH to be significantly correlated with higher cytokine levels and increased disease severity [110].

## Conclusions

In summary, both HLH and severe COVID-19 are hyperinflammatory syndromes characterized by hypercytokinemia and share similar clinical and biochemical profiles. An expanded review, on the other hand, reveals the absence of specific clinical and laboratory HLH diagnostic criteria in severe COVID-19 illness. The absence of these criteria, included in the H-score, resulted in a low score in the majority of severe COVID-19 patients, making an HLH diagnosis unfavorable and distinguishing it from severe COVID-19 disease. Although rare, HLH occurrence in COVID-19 is still noticeable, and the use of H-score in severe COVID-19 patients to identify HLH development in these patients is recommended. The pathogenesis of HLH in COVID-19 patients is multifactorial and mainly attributed to the immune dysregulation caused by COVID-19, in addition to the possible role of gene variants involved in F-HLH and primary immunodeficiency disease.
